# The National Cardiothoracic Centre, Accra Ghana: Proceedings of the second International Update Course in Cardiology - improving the coverage of cardiology services

**Published:** 2012-01-16

**Authors:** Kow Entsua-Mensah, Alfred Doku, Innocent Adzamli

**Affiliations:** 1 National Cardiothoracic Centre, Korle-Bu Teaching Hospital, P. O. Box KB 846, Korle-Bu, Accra, Ghana; 2 Walter Sisulu Paediatric Cardiac Centre for Africa, Sunninghill Hospital, Cnr. Witkoppen & Nanyuki Rd., Sunninghill Park, Sandton, Johannesburg, South Africa

**Keywords:** Cardiovascular disease, double burden of disease, noncommunicable disease, lifestyle modification

## Abstract

Many developing countries now face the growing phenomenon of the double burden of disease. Most are still grappling with infectious diseases resulting from poor environmental sanitation and lack of access to good drinking water like malaria, cholera, and enteric fever. At the same time changes in diet and lifestyle in general in these countries is resulting in increasing numbers of people with obesity, sedentary life styles, increased salt intake from fast foods, increased smoking and consumption of alcohol and fizzy drinks, hypertension and diabetes. To increase the scope and depth of cardiovascular care in Ghana, the National Cardiothoracic Centre, (NCTC), organised the 2^nd^ International Update Course in Cardiology for cardiologists and general practitioners, with emphasis on a practical approach to cardiology. Post conference evaluation indicated that the course was very useful especially for practitioners in district and regional hospitals. Close to 98% of the participants revealed that the update course will greatly impact positively on their management of cardiovascular diseases.

## Introduction

Many low-and middle-income countries are now facing a “double burden” of disease. Non-communicable diseases, which hitherto affected the minority of the indigenous population, are now imposing a growing burden upon low and middle-income countries, which have limited resources and are still struggling to meet the challenges of existing problems with infectious diseases. While they continue to deal with the problems of infectious disease and under-nutrition, they are experiencing a rapid upsurge in non-communicable disease risk factors such as obesity and overweight, particularly in urban settings. It is not uncommon to find under-nutrition and obesity existing side-by-side within the same country, the same community and the same household [1].

The National Cardiothoracic Centre (NCTC), in Accra, Ghana, commenced operations in 1989 and was officially commissioned on 2^nd^ April 1992 [2].

It has since been the foremost centre for the investigation and management of cardiovascular diseases not only in Ghana but also in the West African sub-region. It has also grown to become the hub for the training of cardiovascular surgeons, cardiologists and anaesthetists [3].

These and other training programmes of the Ghana College of Physicians and Surgeons and the West African College of Physicians have helped to increase the stock of trained cardiovascular care givers, but the deficit remains enormous [3]. Cardiovascular care givers are in short supply even in advanced jurisdictions [4]. The situation in Ghana and the sub-region is precarious; against the backdrop of an ever increasing population requiring these services. This calls for innovative training programmes to cater for the short, medium and long term needs of the population [4]. The need to train and incorporate non-cardiologists into the corp of cardiovascular caregivers cannot be overemphasized. The NCTC has therefore identified this strategy as a viable short and medium term option to improve the diagnosis and management of cardiovascular diseases (CVD) in Ghana. To this end, training symposia on the diagnosis and management of common cardiovascular diseases have been held since 2008 on a regular basis.

The 2010 meeting was attended by over 600 physicians and paramedics in Accra and Kumasi. Participants were drawn from Ghana, Sierra Leone, Nigeria and South Africa.

This communication focuses on the 2^nd^ International Update Course in Cardiology organized by the NCTC in collaboration with the Ghana Society of Hypertension and Cardiology and the Ghana College of Physicians and Surgeons.

## The 2011 Symposium

The conference was held from May 24 to 27, 2011, at the office and conference complex of the Ghana College of Physicians and Surgeons in Accra.

### Faculty

Invited speakers were consultants and senior specialists from the National Cardiothoracic Centre, the various teaching hospitals in Ghana and from Nigeria, South Africa and Sierra Leone.

### Participation

A little over two hundred (200) participants, made up of specialists and general practitioners from public and private hospitals across the country as well as from Nigeria were present. Special parallel “breakaway sessions” (workshops) were also held for attending general practitioners, nurses, pharmacists and emergency medical technicians. This conference was unique in that it was not a forum for the presentation of large series and research findings, but rather a “practical approach to training in cardiology for non-cardiologists” was adopted, focusing on the peculiarities of the sub-region, and how cardiologists and non-cardiologists can give quality cardiovascular care to the wider population. It also focused on early risk factor detection and aggressive lifestyle modification, the so called primary prevention, as well as secondary prevention. Very stimulating discussions followed the presentations at each session.

### General organization

Each day of the four day event was characterised by three sessions of three lectures each and a fourth session on practical hands-on cardiovascular training. Special sessions were organised for attending nurses, pharmacists and paramedics on techniques for blood pressure measurement, basic ECG and interpretation, an arrhythmia clinic, cardiopulmonary resuscitation, cardiac intervention procedures and imaging in cardiovascular disease.

Day one saw a discussion on normal blood pressure, control of high blood pressure, treatment targets and resistant hypertension. The next session was dedicated to diabetes mellitus, dyslipidaemia and the management of diabetes in relation to CVD. New and emerging risk factors such as Inflammatory markers, haemostasis thrombotic markers, lipid factors, infectious agents such as CMV, Chlamydia pneumonia, Helicobacter pylori, Herpes simplex virus and other factors such as homocysteine, microalbumin, insulin resistance and the angiotensin converting enzyme-1 (ACE-1) genotype were discussed. Lifestyle modification and sex and the heart ended the presentations for the day. The key lessons for day one were the need to look out for CVD risk factors in all patients irrespective of presenting problems and aggressive management of these risk factors to reduce the incidence of cardiovascular events in the society.

Discussions for day two included atherosclerosis, investigation of ischaemic heart disease, the acute coronary syndrome, acute myocardial infarction and antiplatelet therapy. This was followed by a session on intervention where percutaneous coronary interventions, coronary artery bypass graft and the management of acute arterial occlusions were discussed. Key lessons learned on day two were the need for early recognition of symptoms of CVD and the necessity for early referral to appropriate centres for investigation and possible intervention.

The discussions on day three focused on heart failure in children, diastolic dysfunction, dilated cardiomyopathy and chronic heart failure. Arrhythmias and device interventions in heart failure featured prominently. Rheumatic heart disease, infective endocarditis and pericardial disease ended the day's discussions. These sessions aimed to increase the index of suspicion especially for the non-cardiologists to facilitate early initiation of therapy and appropriate referral and intervention.

On the fourth and last day, emphasis shifted from the heart to some target organs. Deep vein thrombosis, pulmonary embolism, anticoagulation and IVC filter management were discussed. Chronic kidney disease, cardiorenal anaemia syndrome, vertebrobassilar insufficiency, subarachnoid haemorrhage and cerebrovascular accidents in general concluded the discussions. Key lessons for the fourth day were the need for appropriate and timely intervention should secondary end organ damage occur.

### Evaluation

The course was evaluated by the participants using a structured questionnaire for each day's activity. Each activity was rated extremely useful, fairly useful and not useful. After analysis of the retrieved questionnaires, 98% of the participants rated the workshop sessions as being extremely useful. Assessment of the topics such as risk assessment in cardiovascular diseases, blood pressure control and targets, myocardial infarction, chronic heart failure, arrhythmias, rheumatic heart disease and infective endocarditis were rated to be extremely useful by close to 95% of the participants. 94% of participants admitted that the course will impact positively on their future management of cardiovascular diseases.

## Conclusion

Cardiovascular disease is imposing a growing burden on developing countries with limited resources and personnel to manage these conditions, especially in Africa. Training of non-cardiologists to offer improved cardiology service is one strategy adopted by the National Cardiothoracic Centre in Accra. At the end of the four-day symposium, participants found the training extremely useful. We wish to recommend this strategy as a viable option for improving the reach of quality cardiology care in Africa.
